# Case Report: Esthesioneuroblastoma Involving the Optic Pathways

**DOI:** 10.3389/fsurg.2022.875881

**Published:** 2022-04-19

**Authors:** Vithor Ely B. da Silva, Samuel R. Buniatti, Felipe D'Almeida Costa, Mauricio R. Torrecillas, Jean G. de Oliveira, Marcio S. Rassi

**Affiliations:** ^1^Department of Neurosurgery, AC Camargo Cancer Center, São Paulo, Brazil; ^2^Department of Pathology, AC Camargo Cancer Center, São Paulo, Brazil; ^3^Centro de Ensino Superior de Maringá, Maringá, Brazil

**Keywords:** esthesioneuroblastoma, ectopic tumor, cranio-orbito-zygomatic approach, skull base, olfactory neuroblastoma/esthesioneuroblastoma

## Abstract

Olfactory neuroblastoma, or esthesioneuroblastoma, is an uncommon malignant tumor originating from the neural crest that commonly occurs in the upper nasal cavity. Its ectopic origin is extremely rare, especially when located in the optical pathways. This paper reports the case of a giant ectopic esthesioneuroblastoma of the optic pathways that were surgically treated through a cranio-orbital-zygomatic (COZ) craniotomy with extensive resection, in addition to a literature review. The patient is a 46-year-old female presenting with a 4-month history of visual loss in the left eye. Since she was previously blind in the right eye from a traumatic injury, it was evolving to loss of bilateral vision. Imaging depicted an expansive infiltrating lesion involving the entire path of the right optic nerve, extending to the optic chiasm, cisternal portion of the left optic nerve, bilateral optic tract, and hypothalamus. Investigation of pituitary function was unremarkable. Esthesioneuroblastoma is a rare tumor with poorly defined standard clinical management. Its ectopic presentation makes the diagnosis even more challenging, making it difficult to manage these cases properly. Surgeons should be aware of this rare possibility, as early aggressive treatment is likely to be associated with better results.

## Background

Olfactory neuroblastoma, or esthesioneuroblastoma, is a malignant rare tumor that usually occurs in the upper aspect of the nasal cavity due to its origin from the olfactory neuroepithelium with neuroblastic differentiation (1). It frequently extends from the upper part of the nasal cavity to the upper part of the septum, the upper nasal conchae, the roof of the nose, and the cribriform plate of the ethmoidal sinus. Those located outside this region, where the olfactory neuroepithelium does not normally exist, have been reported as ectopic (2). Its ectopic origin is extremely rare, especially when located in the optical pathways.

We report the case of giant ectopic esthesioneuroblastoma that extended along the optic nerve, leading to visual loss, and requiring neurosurgical treatment.

## Clinical Presentation

**Figure 1:**
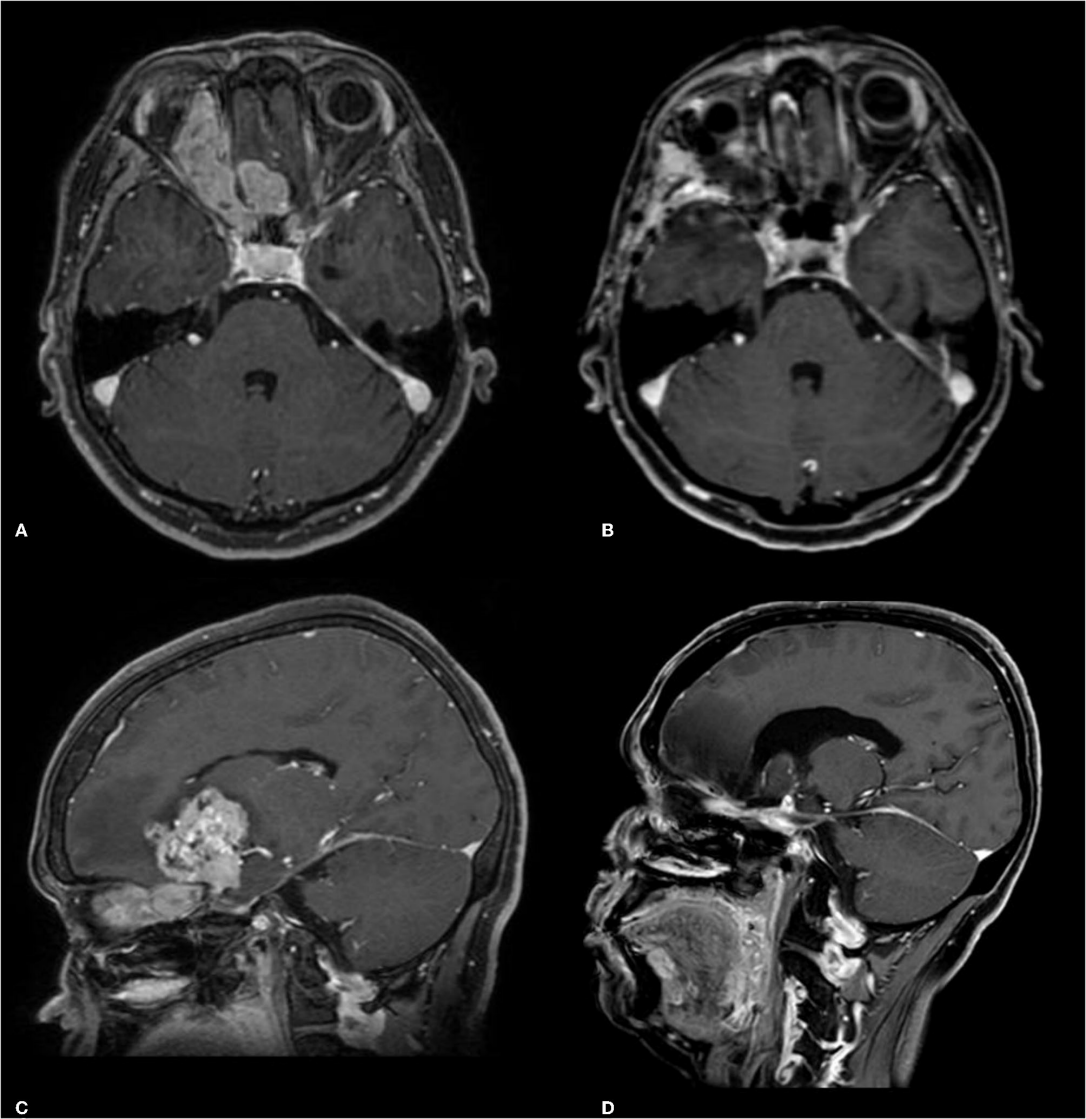
Preoperative post-contrast T1-weighted MRI **(A,C)** and postoperative images **(B,D)**.

The patient is a 46-year-old female presenting with a 4-month history of visual loss in the left eye. Since she was previously blind in the right eye from a traumatic injury, it was evolving to loss of bilateral vision. Imaging depicted an expansive infiltrating lesion involving the entire path of the right optic nerve, extending to the optic chiasm, cisternal portion of the left optic nerve, bilateral optic tract, and hypothalamus (Figure 1). Investigation of pituitary function was unremarkable.

Patient consent was obtained, and surgical removal was performed by the senior author through a cranio-orbital-zygomatic (COZ) approach *via* Transylvanian and pre-temporal routes (Supplementary Video).

**Figure 2:**
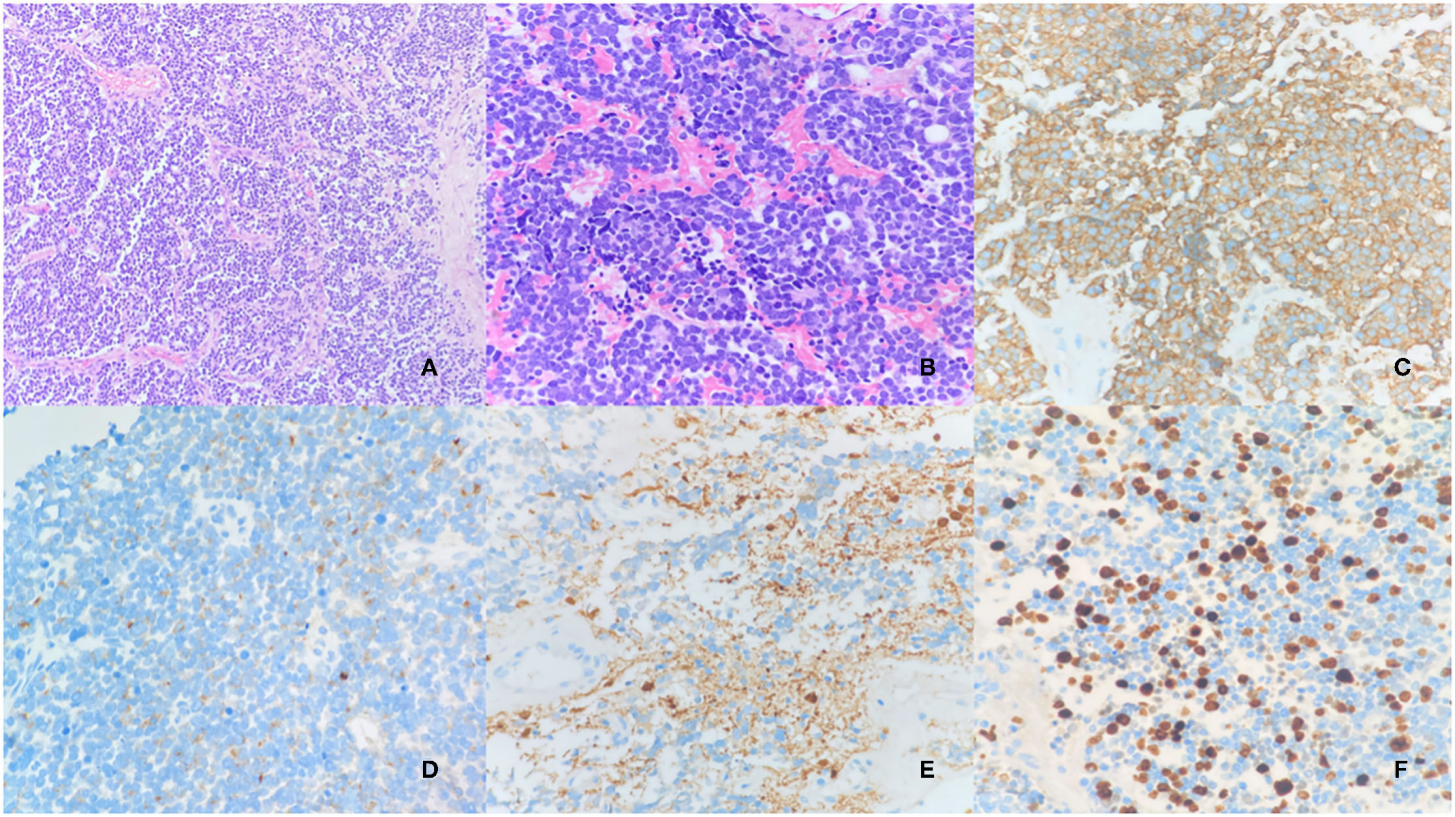
Olfactory neuroblastoma (Hyams III). Proliferation of cells forming lobe sketchs, separated by vascular and hylinized fibrous stroma. HE 10x **(A)**. The cells are hyperchromatic, pleomorphic, and sometimes arranged in gland-like rings or tight annular formations with a true lumen (Flexner–Wintersteiner rosettes). Some mitotic figures can be seen. HE 20x **(B)**. Immunohistochemical stains: Synaptophysin **(C)**; Chromogranin **(D)**; S100: positive in sustentacular periphery cells **(E)**; Ki-67 **(F)**.

After the procedure, the patient showed visual improvement in the left eye, with transient diabetes insipidus on the first postoperative day. Pathology showed an olfactory neuroblastoma grade III of Hyams (Figure 2). Treatment was continued with adjuvant radiotherapy.

Postoperative imaging showed gross total resection, in addition to the absence of metastatic foci along the neuroaxis or lymph node involvement, characterizing a T4N0M0 tumor according to Dulguerov et al. (3), or stage C using the modified Kadish classification (4).

## Discussion

Esthesioneuroblastoma is a rare tumor with sparse data in the literature. It predominantly occurs in young adults, and the age of presentation varies from 40 to 70 years, with men and women equally affected (1). Although the exact cell type and location have not yet been defined, the common assumption is that it is derived from cells in the neural crest of the upper nasal cavity (5).

Clinical symptoms depend on the location and extension of the tumor, and, therefore, nasal symptoms, such as epistaxis, nasal obstruction, and anosmia, are the most common (5). However, other symptoms, such as headache, diplopia, visual loss, and seizures can also occur. Due to its rarity and unusual presentation, ectopic olfactory neuroblastoma becomes a disease that is difficult to suspect, merging with other types of tumors.

Neither of the two clinical staging systems, TNM by Dulguerov et al. or the modified Kadish classification is ideally geared to the staging of ectopic olfactory neuroblastoma. The most critical information seems to be whether the tumor extends to the anterior cranial fossa and orbit and whether it is related to lymphadenopathy, as this has the greatest impact on treatment planning and prognosis (6).

The theory to explain the ectopic origin of these tumors is speculative and is based on the idea that there may be ectopic cell debris during embryologic development (7). The theory supporting the origin of ectopic esthesioneuroblastoma was first suggested by Jakumeit in 1971 and is the theory of the terminal nervous system (8). Embryologically, the olfactory placode is divided into two systems; the first system contains the olfactory nerve and the vomeronasal nerve, and the second system contains the terminal nerve that develops immediately caudal to the first. Both will degenerate into fetal life. The terminal nerve ganglion and neurons spread diffusely across the cribriform plate, nasal septum, nasal mucosa, Bowman's gland, mucosa naris, crista Galli, and hypothalamus. The persistence of these cells beyond fetal life can provide the source of ectopic esthesioneuroblastoma.

Another possible theory, provided by Zappia et al., describes a model of blocked migration of neuronal cells from the olfactory placode that may provide the origin of these tumors in a case of Kallman syndrome, which is a congenital condition defined by the absence of olfactory bulbs and pituitary hypoplasia. Even patients, who do not have Kallman's syndrome, may exhibit less disorderly migration along the pathway, which may progress to an esthesioneuroblastoma in the future (9).

The treatment of esthesioneuroblastoma is controversial, mainly due to its rarity and lack of data in the literature to support therapeutic regimens, with surgical resection followed by postoperative radiotherapy, being the option that showed better treatment results in retrospective reports, compared to isolated radiotherapy, although orbital invasion is associated with adverse survival outcomes (5, 10). The classifications of Kadish, Dulguerov, and Hyams help predict prognosis and guide treatment, with no superiority of any (5). In the case presented, we chose a cranio-orbital-zygomatic craniotomy to allow good brain exposure and provide several routes to the tumor location, including Transylvanian and pretemporal. Adjuvant radiotherapy in the surgical bed complemented the operative treatment in this patient.

The role of chemotherapy in the treatment of these patients is still questionable. Neoadjuvant chemotherapy has been reported to show positive responses in locally advanced cases and appears to play an important role, especially in tumors with difficult resection (5). In the initial stage, some groups advocate platinum-based therapy whenever possible, while other groups postpone chemotherapy treatment. More studies with longer follow-ups are needed to interpret the results.

## Conclusion

Esthesioneuroblastoma is a rare tumor with poorly defined standard clinical management. Its ectopic presentation makes the diagnosis even more challenging, making it difficult to manage these cases properly. Surgeons should be aware of this rare possibility, as early aggressive treatment is likely to be associated with better results.

## Data Availability Statement

The original contributions presented in the study are included in the article/Supplementary Material, further inquiries can be directed to the corresponding author/s.

## Ethics Statement

Written informed consent was obtained from the individual(s) for the publication of any potentially identifiable images or data included in this article.

## Author Contributions

All authors listed have made a substantial, direct, and intellectual contribution to the work and approved it for publication.

## Conflict of Interest

The authors declare that the research was conducted in the absence of any commercial or financial relationships that could be construed as a potential conflict of interest.

## Publisher's Note

All claims expressed in this article are solely those of the authors and do not necessarily represent those of their affiliated organizations, or those of the publisher, the editors and the reviewers. Any product that may be evaluated in this article, or claim that may be made by its manufacturer, is not guaranteed or endorsed by the publisher.

## Supplementary Material

Supplementary Video 1 Cranio-orbito-zygomatic approach for ectopic esthesioneuroblastoma.
